# FunctionaL Assessment Scale of Hemianopia (FLASH): A New Multidisciplinary Tool to Assess Hemianopia in Patients with Severe Acquired Brain Injury

**DOI:** 10.3390/healthcare11212883

**Published:** 2023-11-02

**Authors:** Susanna Lucatello, Sara De Angelis, Concetta Di Lorenzo, Marco Iosa, Luisa Magnotti, Marta Di Paolo, Maria De Luca, Maria Gabriella Buzzi, Marco Tramontano

**Affiliations:** 1 Fondazione Santa Lucia IRCCS, 00179 Rome, Italy; s.lucatello@hsantalucia.it (S.L.); s.deangelis@hsantalucia.it (S.D.A.); t.dilorenzo@hsantalucia.it (C.D.L.); l.magnotti@hsantalucia.it (L.M.); m.deluca@hsantalucia.it (M.D.L.); mg.buzzi@hsantalucia.it (M.G.B.); 2 Department of Psychology, Sapienza University of Rome, 00185 Rome, Italy; 3 Department of Biomedical and Neuromotor Sciences (DIBINEM), Alma Mater University of Bologna, Via Massarenti 9, 40138 Bologna, Italy; marco.tramontano@unibo.it; 4 Unit of Occupational Medicine, IRCCS Azienda Ospedaliero-Universitaria di Bologna, Via Pelagio Palagi 9, 40138 Bologna, Italy

**Keywords:** severe acquired brain injury, visual field, hemianopia, homonymous visual field defects, homonymous hemianopia, spatial neglect, cognitive visual functions, psychometry

## Abstract

Background: Severe acquired brain injury (sABI) encompasses a range of neurological impairments. Visual dysfunction, particularly homonymous visual field defects (HVFDs) and homonymous hemianopia (HH), commonly afflicts sABI survivors, affecting their cognitive and motor rehabilitation. This study presents the FunctionaL Assessment Scale of Hemianopia (FLASH), developed to analyze the most common postural behaviors exhibited by sABI patients with hemianopia during activities of daily living. A comparison to traditional static automated perimetry for diagnosing visual field deficits (VFDs) to determine the sensitivity and specificity of the FLASH was used. Additionally, this study also aimed to assess its reliability. Methods: Fifty-six patients (25 F, 31 M, mean age 60.59 ± 14.53) with strokes in the sub-acute phase (<6 months from the onset) were assessed with both FLASH and a Humphrey Field Analyzer. Results: After removing two items found to be less reliable than others, FLASH showed high sensitivity (81%) and specificity (77%) when compared to static automated perimetry. Inter-rater reliability was also high, with an intra-class correlation coefficient of 0.954, as well as the internal consistency computed by Cronbach’s alpha, equal to 0.874. Conclusion: FLASH could offer a valuable and cost-effective screening tool for VFD in sABI patients during neurorehabilitation, with potential implications for healthcare cost reduction.

## 1. Introduction

Severe acquired brain injury (sABI) can result from various causes such as stroke, anoxia, hypoxia, infections, trauma, or degenerative diseases. It is characterized by severe brain damage, a coma lasting at least 24 h with a Glasgow Coma Scale [1] value equal to or lower than 8, and complex and severe neurological disabilities [2,3]. Individuals with sABI may experience a wide range of temporary or long-term cognitive, motor, and behavioral impairments, significantly impacting their quality of life. Studies have shown that a substantial proportion (30–85%) of sABI survivors experience some form of visual dysfunction [4,5], particularly homonymous visual field defects (HVFDs) resulting from lesions affecting the visual pathways posterior to the chiasm [6]. A common visual impairment is homonymous hemianopia (HH), which affects either the right or left half-field in both eyes and impacts various cognitive visual functions [7,8,9,10]. HVFDs often coexist with other conditions following diffuse axonal damage due to sABI, leading to disconnection syndrome and compromising the brain areas responsible for integrative activities. Consequently, attentional, executive, and memory functions, which underlie cognitive development, learning, and purposeful action, are affected [11]. This complexity makes it challenging to diagnose specific deficits. Therefore, an accurate diagnosis is crucial for developing a personalized rehabilitation program [12]. Static automated perimetry performed with the Humphrey Field Analyzer (The HFA II- I, Humphrey Instruments, Dublin, CA) is the gold standard for diagnosing visual field deficits [13]. In patients with sABI sequelae, the reliability of visual field examination with an automated perimetry device is often compromised due to factors such as attention deficits, mental fatigue, or poor fixation control. Inconsistent responses in the same area of the visual field or difficulties in maintaining fixation render static automated perimetry ineffective [14]. Therefore, it is crucial to develop a reliable tool capable of accommodating variables that might hinder the identification of impairments in individuals with behavioral disorders while also ensuring ease of administration [15].

In light of the above, we have developed a FunctionaL Assessment Scale of Hemianopia (FLASH) to analyze the most common postural behaviors exhibited by sABI patients with hemianopia during activities of daily living.

To the best of our knowledge, there are currently no specific scales designed to identify visual field defects (VFDs) that can impact or influence rehabilitation outcomes during post-acute hospitalization. Our hypothesis is that a functional assessment of hemianopia, as provided by FLASH, can offer a qualitative and structured analysis of the most common behaviors observed in patients with hemianopia.

With this in mind, the primary aim of this study was to evaluate the accuracy of FLASH in comparison to standard automatic perimetry. The secondary aims included assessing the inter-rater reliability, internal consistency, and concurrent validity of the scale.

## 2. Materials and Methods

The study protocol was approved by the Local Ethics Committee of IRCCS Fondazione Santa Lucia (FSL) (CE/PROG.907). Written informed consent was provided by all the enrolled patients or their legally authorized representatives. This study was conducted in accordance with the Declaration of Helsinki and met the Good Clinical Practice standards. All procedures contributing to this work comply with the guidelines for developing and validating a questionnaire in medicine [16].

### Questionnaire Development

An expert committee composed of health professionals (1 neurologist, 1 ophthalmologist, 2 speech therapists, 2 physical therapists, 1 neuropsychologist) and experts of psychometry [17,18,19,20] developed the FLASH on the theoretical concept that a functional assessment of hemianopia could offer a qualitative and structured analysis of the most common behaviors observed in patients with hemianopia The expert committee’s role was to consolidate all the versions of the questionnaire and develop the prefinal version of the questionnaire for field-testing. The committee reached a consensus on any discrepancy.

To develop the questionnaire, the committee (i) carried out an extensive review of the existing literature to understand the current state of knowledge on the condition and the existing assessment tools [21]; (ii) established a clear conceptual framework for the scale, which includes understanding its functional impact on patients and clinicians and determining the key domains or aspects of life affected by this condition; (iii) identified the dimensionality of the construct and assigned the same weight to the question. It was designed to be administered by clinical staff, and close-ended items were chosen. A preliminary version of the scale was then administered to a small group of patients with hemianopia to assess its feasibility, clarity, and relevance. Experts in the field, such as neurologists, ophthalmologists, rehabilitation specialists, and other relevant professionals, were consulted to review and provide feedback on the generated items.

The selection of items for the FLASH scale considered the most common symptoms experienced by patients with hemianopia during their daily activities [22,23] and their functional mechanisms to compensate for the hemianopia [24].

Prior to the validation study, the scale underwent rigorous external assessment. The FLASH was passed through 2 external assessors and the author group 2 times to refine and enhance its content. Feedback received was carefully considered and included if it improved the scale’s comprehensiveness and relevance.

The first part of the FLASH includes demographic and clinical data. In the second section, information about the position of the head, the patient’s spontaneous gaze direction, and the alignment of binocular fixation is recorded, reporting specific scores [24]. During the initial assessment, the correct alignment of the visual axes is determined. If the alignment is not correct, it is necessary to identify whether the fixation is rotated or inclined based on the direction of gaze. Detailed guidance on this process is provided in the FLASH User Guide (See Supplementary Materials) to ensure standardized assessment criteria and accurate identification of the presence or absence of target behaviors. This guide aims to facilitate consistent usage of the scale.

The collection of these data is carried out during the initial patient analysis (specific orthoptics tests are not proposed; instead, it relies on a qualitative investigation).

The third section of the scale addresses behaviors that might arise during activities conducted in a neurorehabilitation setting. For example, certain items assess whether the patient employs head compensation strategies (e.g., tilting and/or rotating the head to explore the impaired visual field) or consistently omits a portion of space (either to the right or left) during different tasks, such as writing. There are also items related to spatial management during walking or moving with a wheelchair, including deviations in trajectory, collisions with obstacles or people, and whether the patient is surprised by sudden appearances of people or objects on the affected side.

Each item allows for indicating whether the phenomenon is present, absent, or not assessable (e.g., if the patient has not yet achieved motor autonomy to move independently, the corresponding item will be marked as not assessable).

Static visual field testing was conducted by an experienced orthoptist on all enrolled patients using a Humphrey Field Analyzer (HFA) 3 (Zeiss Meditech Jena, Germany). The test was performed separately for the left and right eye. The VFD was classified as right or left hemianopia, right or left (superior or inferior) quadrantanopia, no deficit, not reliable result, or other deficits.

Simultaneously, the FLASH scale was administered by rehabilitation professionals, including speech and physical therapists, who had received specific training on using the FLASH (with at least 10 h of training).

The assistance of a caregiver was used to complete the FLASH, not for administering the scale itself but to obtain a comprehensive understanding of the patient’s behaviors. This understanding extended beyond rehabilitation sessions, such as speech therapy and neuromotor therapy, and included activities in the patient’s daily life.

The therapists who completed the FLASH were blind to the results of the static automated perimetry assessment.

The sample size was determined based on the study by Lemke and colleagues [13] which investigated the feasibility of automated perimetry in patients with acquired brain injury and reported the need for at least 48 subjects to obtain reliable results. A consecutive convenience sampling method was employed.

The inclusion criteria were as follows: time from sABI <6 months; age ≥ 18 years; ischemic or hemorrhagic lesion resulting from a stroke or diffuse axonal injury following traumatic brain injury; and ability to understand verbal commands (Level of Cognitive Functioning (LCF) ≥ 7 [25,26]).

The exclusion criteria were as follows: cognitive deficits affecting the ability to understand task instructions (Mini-Mental State Examination > 24), severe unilateral spatial neglect (diagnosed with a test battery that included the Letter Cancellation test, Barrage test, Sentence Reading test, and the Wundt–Jastrow Area Illusion Test), presence of other neurological and psychiatric diseases, history of drug and alcohol addiction, or repeated sABI. After patients were admitted to the Neurorehabilitation Unit, fifty-eight inpatients with a diagnosis of sABI (with or without revealed hemianopia at the neurological assessment) met the inclusion criteria and were enrolled in this study using a consecutive sampling approach from November 2020 to October 2022. The FLASH scale was completed for each patient.

All statistical analyses were conducted using the IBM Statistical Package for the Social Sciences (SPSS), version 22. First, we performed an item analysis to evaluate the discriminative validity of each item. This analysis examined how the scores for each item relate to different HFA findings using the chi-squared test. Secondly, we conducted an analysis of internal consistency using Cronbach’s alpha and assessed inter-rater reliability using the Intraclass Correlation Coefficient, ICC (2.1), with a two-way random effects model. The 95% confidence interval (95%CI)) was also reported. In accordance with the statistical literature, values of ICC (2.1) and alpha > 0.7 were considered indicative of good reliability. Any items found to be invalid or unreliable were excluded, and the total score of the scale was re-calculated only based on valid and reliable items. Next, the total score was used to assess the concurrent validity in relation to the findings of the HFA, using the Kruskal–Wallis analysis. Finally, we employed a Receiver Operating Characteristic (ROC) curve to evaluate the diagnostic ability of the FLASH score, which was used as a classifier. The ROC curve was generated by computing the area under the curve (AUC) with the objective of determining the best threshold for the FLASH to provide an accurate diagnosis of hemianopia. This process optimized sensitivity and specificity using the Youden index (Y).

## 3. Results

Fifty-six patients (25 females, 31 males, mean age 60.59 ± 14.53 years) out of the initial fifty-eight, were included in this study. Among them, 32 had ischemic strokes, 16 experienced hemorrhagic strokes, and 8 had severe TBI (Glasgow Coma Scale ≤ 8). All these patients were in the sub-acute phase (<6 months from acute event). They underwent assessments using both the FLASH and the HFA. Two patients dropped out of the study for reasons unrelated to the research.

The HFA-based assessment identified the following results among the patients: 16 patients had no deficits, 4 patients had quadrantanopia, 21 patients had hemianopia, 9 patients had other deficits, and in 6 cases, the test did not yield reliable results for diagnosing the condition.

### 3.1. Item Analysis

The inter-rater reliability assessment for each item demonstrated an agreement of 100% (*p* < 0.001) for all items, except for item C (86.7%, *p* = 0.011) and item I (90.9%, *p* = 0.006). To assess the concurrent validity of each item with respect to the diagnosis obtained by the HFA, an item response analysis was conducted using a chi-squared test. Significant differences in scores according to the diagnosis were found for items B (*p* = 0.011), D (*p* < 0.001), E (*p* < 0.001), F (*p* < 0.001), G (*p* = 0.041), L (*p* = 0.039), and M (*p* = 0.019) but not for items A (*p* = 0.426), C (*p* = 0.292), H (*p* = 0.258), and I (*p* = 0.078).

The Cronbach’s alpha was 0.874, and it could be improved by removing item A (0.888) or item C (0.886). Removing item H or item I did not improve the Cronbach’s alpha (0.855, and 0.851, respectively). However, by removing both items A and C, the Cronbach’s alpha increased to 0.904.

The inter-rater reliability was measured to be ICC = 0.903 (95%CI: 0.736–0.966). After removing items A and C, it increased to ICC = 0.954 (0.868–0.984). Therefore, based on these findings, items A and C were removed from the scale, while items H and I remained. In the subsequent analyses, the total score of the FLASH scale was computed using the following items: B, D, E, F, G, H, I, L, M.

### 3.2. Total Score Analysis

**Figure 1:**
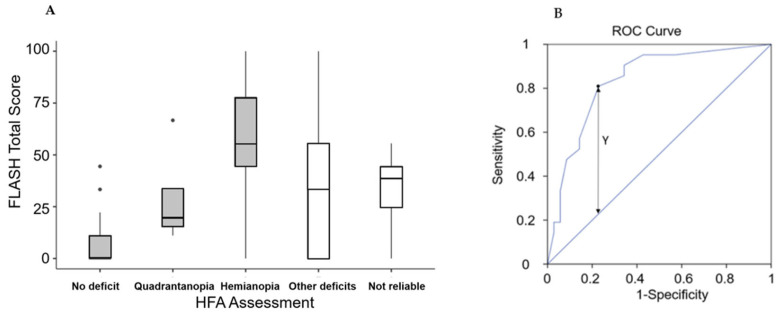
(**A**) Box and whiskers plot of the FLASH total score (boxes represent first and third quartiles, with the bold lines representing the median values; the lines report the range between minimum and maximum of the values within 1.5 times the interquartile range; the points out of this range are represented by circles). (**B**) Receiver Operating Characteristic (ROC) curve of FLASH total score, with the identification of the cut-off (FLASH = 39) obtained by using the Y-index.

The analysis of concurrent validity was conducted using an analysis of variance to determine if the total score significantly differed among the five possible diagnoses from the HFA (no deficit, not reliable result, quadrantanopia, other deficits, hemianopia). Descriptive results are presented in Figure 1A, and there were significant differences in the total scores for the five diagnoses (χ^2^ = 18.8, *p* < 0.001). Subsequently, an ROC analysis was performed to identify the FLASH cut-off point for diagnosing hemianopia. The area under the curve was AUC = 0.836. An optimal cut-off point (Y = 0.581) was found for FLASH score = 39, and at this value, the proposed scale demonstrated a sensitivity of 81% and a specificity of 77% (Figure 1B).

## 4. Discussion

This study aimed to assess the accuracy (sensitivity and specificity) of a new functional clinical scale, the FLASH, in comparison to the standard static automated perimetry for detecting visual field deficits (VFD). Secondarily, we evaluated its reliability. The results indicated that the FLASH, after the removal of two non-reliable items, displayed high levels of sensitivity and specificity when compared to static automated perimetry. When static automated perimetry detected hemianopia, the FLASH identified distinct behaviors associated with VFD, such as compensatory head movements or signs of surprise when exposed to objects. In contrast, when a participant had an intact visual field, the FLASH accurately recorded the absence of these behaviors.

This study suggests that the FLASH could serve as an innovative and non-invasive preliminary approach to detect VFD without the need for instrumental assessments. While most patients examined in this study exhibited VFD, a percentage of participants (11%) yielded unreliable results with static automated perimetry, primarily due to comorbid deficits [27]. This underscores the importance of having alternative tools to accurately identify and classify VFD, particularly when interpreting static automated perimetry results becomes challenging.

This study introduced the FLASH as a tool that allows rehabilitation professionals to recognize and emphasize specific behaviors indicative of VFD. This approach facilitates accurate assessments during post-acute hospitalization and enables the development of customized rehabilitation interventions for patients. The analysis of inter-rater reliability showed that different professionals’ observations of the same subject were consistent, promoting interdisciplinary collaboration and a multidisciplinary approach to patient care. The sensitivity and specificity of the FLASH were reported as 81% and 77%, respectively, which aligns with previous studies on qualitative examinations such as the confrontation method of visual field tests [28,29]. The sensitivity and specificity values are known to depend on the type, density, and cause of the visual field defect, as well as the specific confrontation method employed. Kerr and colleagues [30] reported that the highest levels of sensitivity and specificity were 74% and 93%, respectively, when confrontation was performed.

Moreover, the results from using the FLASH are relatively cost-effective for clinical practice. The application of FLASH could streamline referrals to orthoptists and potentially reduce the necessity for expensive diagnostic tests like static automated perimetry, particularly in cases where patients may lack the cognitive-motor abilities to reliably complete such tests. Overall, this study suggests that the FLASH shows promise as a tool for detecting VFD, offering advantages such as easy administration, accurate observation of specific behaviors, inter-rater reliability, and potential cost savings in clinical practice.

The data analysis did not reveal a correlation between postural disturbances and hemianopia in the current sample of patients. This could be attributed to the presence of postural alterations that are characteristic of these patients, making it challenging to differentiate specific postural compensations, especially during the post-acute phase. Factors such as spasticity of the sternocleidomastoid muscle or the use of cervical collars in patients with cervical spine fractures can contribute to postural difficulties and complicate the assessment of postural disturbances related to hemianopia. Another contributing factor could be monocular vision due to the ocular aberrations. In cases of significant visual alterations, the central nervous system may not effectively process the information from one eye, resulting in reduced or abolished postural compensation. Patients with hemianopia often exhibit other ophthalmological–orthoptic disorders, such as strabismus, ocular muscle hypofunction, or gaze paralysis, further complicating the clinical evaluation.

Future studies should consider evaluating patients at a later point or include patients in the chronic phase to assess the sensitivity and specificity of the remaining items in the FLASH. This would allow for the investigation of postural disturbances and specific compensations related to hemianopia after improvement or stabilization of neuromotor sequelae. Indeed, assessing patients with greater motor abilities could be beneficial in evaluating items that were not significant in the current study, such as obstacles encountered while maintaining a correct head/gaze position or difficulties in moving through crowded areas.

Our study has some limitations. The first limitation pertains to the sample size. We included only 56 patients based on the sample size determined in a prior study [13], and we used a consecutive convenience sample. Despite the relatively small sample, it was adequate to yield statistically significant results, which may be further substantiated by enrolling a larger sample in future studies. Another important limitation is that the scale was administered in the Italian language and we did not conduct a transcultural adaptation in English.

Furthermore, future studies should investigate the comparison with traditional visual field testing and include more patients who are unable to perform HFA to gain a more comprehensive understanding of the tool’s utility.

## 5. Conclusions

These results suggest that FLASH could be a valuable tool for screening VFD in patients with sABI during neurorehabilitation hospitalization, with strong inter-rater reliability among healthcare professionals. It provides objective and beneficial functional observations for assessing hemianopia. Administering the FLASH can provide additional relevant information that enhances the accuracy of the clinical decision-making process, facilitating the identification of patients who may require urgent referrals for instrumental perimetry.

## Data Availability

Data are available upon reasonable request to the corresponding author.

## Supplementary Materials

The following supporting information can be downloaded at: https://www.mdpi.com/article/10.3390/healthcare11212883/s1, FunctionaL Assessment Scale of Hemianopia (FLASH) and user guide for the FLASH.

## References

[1] Teasdale G., Maas A., Lecky F., Manley G., Stocchetti N., Murray G. (2014). The Glasgow Coma Scale at 40 years: Standing the test of time. Lancet Neurol..

[2] (1998). Linee Guida del Ministero della Sanità per le Attività di Riabilitazione. Gazzetta Ufficiale della Repubblica Italiana, Serie Generale n° 124 del.

[3] Silvestro D., Mazzetti M., Melia C., Stagno M.T., Carlesimo G.A., Bivona U., Formisano R. (2017). Educational action in the rehabilitation of severe acquired brain injuries: The role of self-awareness. Ann. Dell’istituto Super. Sanita.

[4] Khan S., Leung E., Jay W.M. (2008). Stroke and visual rehabilitation. Top. Stroke Rehabil..

[5] Kerkhoff G. (2000). Neurovisual rehabilitation: Recent developments and future directions. J. Neurol. Neurosurg. Psychiatry.

[6] Goodwin D. (2014). Homonymous hemianopia: Challenges and solutions. Clin. Ophthalmol..

[7] Karnath H.O., Rennig J., Johannsen L., Rorden C. (2011). The anatomy underlying acute versus chronic spatial neglect: A longitudinal study. Brain A J. Neurol..

[8] Obuchowska I., Mariak Z. (2012). Homonymous hemianopsia. Klin. Ocz..

[9] Martinelli F., Perez C., Caetta F., Obadia M., Savatovsky J., Chokron S. (2020). Neuroanatomic correlates of visual hallucinations in poststroke hemianopic patients. Neurology.

[10] Zhang X., Kedar S., Lynn M.J., Newman N.J., Biousse V. (2006). Natural history of homonymous hemianopia. Neurology.

[11] McKinlay A. (2010). Controversies and outcomes associated with mild traumatic brain injury in childhood and adolescences. Child Care Health Dev..

[12] Pouget M.C., Lévy-Bencheton D., Prost M., Tilikete C., Husain M., Jacquin-Courtois S. (2012). Acquired visual field defects rehabilitation: Critical review and perspectives. Ann. Phys. Rehabil. Med..

[13] Lemke S., Cockerham G.C., Glynn-Milley C., Lin R., Cockerham K.P. (2016). Automated Perimetry and Visual Dysfunction in Blast-Related Traumatic Brain Injury. Ophthalmology.

[14] Gangeddula V., Ranchet M., Akinwuntan A.E., Bollinger K., Devos H. (2017). Effect of Cognitive Demand on Functional Visual Field Performance in Senior Drivers with Glaucoma. Front. Aging Neurosci..

[15] Suchoff I.B., Kapoor N., Ciuffreda K.J., Rutner D., Han E., Craig S. (2008). The frequency of occurrence, types, and characteristics of visual field defects in acquired brain injury: A retrospective analysis. Optometry.

[16] Tsang S., Royse C.F., Terkawi A.S. (2017). Guidelines for developing, translating, and validating a questionnaire in perioperative and pain medicine. Saudi J. Anaesth..

[17] Iosa M., Galeoto G., De Bartolo D., Russo V., Ruotolo I., Spitoni G.F., Ciancarelli I., Tramontano M., Antonucci G., Paolucci S. (2021). Italian Version of the Pittsburgh Rehabilitation Participation Scale: Psychometric Analysis of Validity and Reliability. Brain Sci..

[18] Tramontano M., Cascioli S., Magnotti L., Sovani M., Gaita A., Galeoto G., Berardi A., Valente D., De Angelis S., Salvia A. (2023). Therapeutic educational workshops for caregivers of patients with severe acquired brain injury. La Clin. Ter..

[19] Berardi A., Graziosi G., Ferrazzano G., Casagrande Conti L., Grasso M.G., Tramontano M., Conte A., Galeoto G. (2022). Evaluation of the Psychometric Properties of the Revised Piper Fatigue Scale in Patients with Multiple Sclerosis. Healthcare.

[20] Bivona U., Ferri G., De Luca M., Lucatello S., Aloisi M., Contrada M., Ciurli P., Bandiera V., Lo Sterzo P., Lombardi G. (2022). Self-Awareness Multilevel Assessment Scale (SAMAS): Psychometric analysis of inter-rater reliability. Ann. Dell’istituto Super. Sanita.

[21] Helboe K.S., Eddelien H.S., Kruuse C. (2023). Visual symptoms in acute stroke—A systematic review of observational studies. Clin. Neurol. Neurosurg..

[22] de Haan G.A., Heutink J., Melis-Dankers B.J., Brouwer W.H., Tucha O. (2015). Difficulties in Daily Life Reported by Patients With Homonymous Visual Field Defects. J. Neuro-Ophthalmol. Off. J. N. Am. Neuro-Ophthalmol. Soc..

[23] Mennem T.A., Warren M., Yuen H.K. (2012). Preliminary validation of a vision-dependent activities of daily living instrument on adults with homonymous hemianopia. Am. J. Occup. Ther. Off. Publ. Am. Occup. Ther. Assoc..

[24] Alwashmi K., Meyer G., Rowe F.J. (2022). Audio-visual stimulation for visual compensatory functions in stroke survivors with visual field defect: A systematic review. Neurol. Sci. Off. J. Ital. Neurol. Soc. Ital. Soc. Clin. Neurophysiol..

[25] Gouvier W.D., Blanton P.D., LaPorte K.K., Nepomuceno C. (1987). Reliability and validity of the Disability Rating Scale and the Levels of Cognitive Functioning Scale in monitoring recovery from severe head injury. Arch. Phys. Med. Rehabil..

[26] Ciurli P., Bivona U., Barba C., Onder G., Silvestro D., Azicnuda E., Rigon J., Formisano R. (2010). Metacognitive unawareness correlates with executive function impairment after severe traumatic brain injury. J. Int. Neuropsychol. Soc..

[27] Bahnemann M., Hamel J., De Beukelaer S., Ohl S., Kehrer S., Audebert H., Kraft A., Brandt S.A. (2015). Compensatory eye and head movements of patients with homonymous hemianopia in the naturalistic setting of a driving simulation. J. Neurol..

[28] Harvey R., Jellinek H. (1981). Functional performance assessment: A program approach. Arch. Phys. Med. Rehabil..

[29] De Luca M., Baroncini M., Matano A., Di Lorenzo C., Magnotti L., Lucatello S., Mulas M., Pollarini V., Ciurli M.P., Nardo D. (2023). Sensitivity and Specificity of the Brentano Illusion Test in the Detection of Visual Hemi-Field Deficits in Patients with Unilateral Spatial Neglect. Brain Sci..

[30] Kerr N.M., Chew S.S., Eady E.K., Gamble G.D., Danesh-Meyer H.V. (2010). Diagnostic accuracy of confrontation visual field tests. Neurology.

