# Supplementary material for: FunctionaL Assessment Scale of Hemianopia (FLASH): A New Multidisciplinary Tool to Assess Hemianopia in Patients with Severe Acquired Brain Injury
Source: Healthcare (Basel). 2023 Nov 2;11(21):2883. doi: 10.3390/healthcare11212883 (PMC10647452; doi:10.3390/healthcare11212883)
Supplement: Supplementary file 1 [file healthcare-11-02883-s001.zip › healthcare-2626379-supplementary.pdf]

Functional Assessment Scale of Hemianopia (FLASH)

Date

_____/_____/_____

Name: ______________________________

Surname: ______________________________

Etiology: ______________________________

Professional (name and surname)

____

QUALITATIVE CLINICAL OBSERVATION

**a.** Spontaneous gaze direction	☐ center	☐ right	☐ left		
**b.** Abnormal Head Position	☐ yes rotated RH	☐ yes rotated LH	☐ yes inclinated RH	☐ yes inclinated LH	☐ no
**c.** Alignment of binocular fixation	☐ central	☐ rotated RH	☐ rotated LH	☐ inclinated RH	☐ inclinated LH

User signature

____

Functional Assessment Scale of Hemianopsia (FLASH)

REHABILITATION SETTING

**d.** Utilizing head compensation strategies in desk task performance	☐ yes	☐ no	☐ N.A.
**e.** If yes:			
- When exploring a hemifield (right/left)	☐ yes (specify which one) ______	☐ no	☐ N.A.
- When exploring only a portion of the hemifield (e.g., lower right quadrant)	☐ yes (specify which one) ______	☐ no	☐ N.A.
**f.** In tasks such as writing, he/she often begins to employ compensation strategies, even when prompted, and consistently neglects a specific portion of space, whether it is on the right-hand side or left-hand side	☐ yes	☐ no	☐ N.A.
**g.** He/she encounters obstacles while maintaining the correct position of his/her head and/or gaze	☐ yes	☐ no	☐ N.A.
**h.** Deviation from the wheelchair's intended path or guide is observed.	☐ yes	☐ no	☐ N.A.
**i.** He/she experiences difficulty and discomfort when moving in crowded areas due to encountering people and various obstacles.	☐ yes	☐ no	☐ N.A.
**l.** He/she rotates his/her head and directs his/her gaze toward the reduced hemifield, and sometimes appears surprised when he/her identifies a person or object that is pointed out to him/her	☐ yes	☐ no	☐ N.A.
m. He/her often appears surprised by the sudden appearance of objects or people in his/her vicinity.	☐ yes	☐ no	☐ N.A.

ADDITIONAL NOTES

____

Functional Assessment Scale of Hemianopsia (FLASH) - User Guide

The scale consists of two main sections. The first part involves the registration of personal and clinical patient information, such as their name, surname, and the etiology of their disease, along with the observation recording date.
The second section is dedicated to qualitative clinical observations. It encompasses data that becomes evident during the initial examination of the patient when they first come under our observation. This section includes observations related to the patient's head position, their spontaneous gaze direction, and the presence or absence of gaze deviation. No specific tests are proposed; instead, we rely on qualitative observation. For instance, in the 'spontaneous gaze direction' sub-section, we record the direction of the patient's gaze in their baseline condition, without any external stimulations.

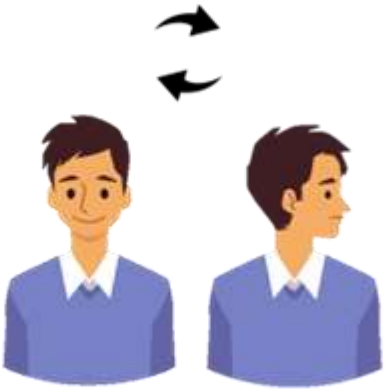

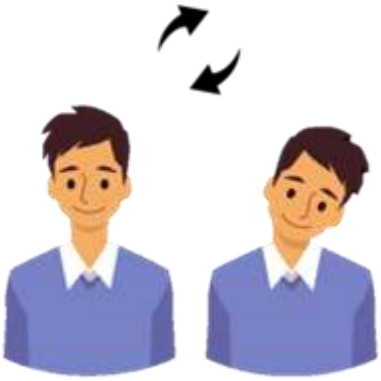

An 'Abnormal head position' refers to a compulsory head orientation caused by abnormalities affecting the visual system. This condition is indicated by the presence of any head rotation or inclination. Below, there are two images that can help distinguish between these different postural conditions: rotation (Figure 1) and inclination (Figure 2).

Figure 2 - Inclination *Figure 1 - Rotation*

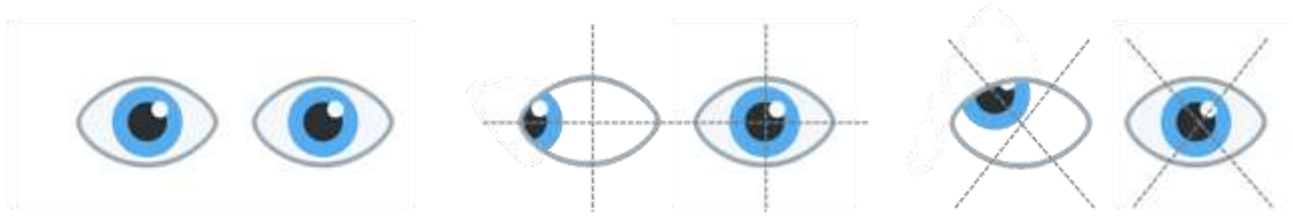

In the 'alignment of binocular fixation' section, 'central fixation' will be noted when the visual axes are correctly aligned (see Figure 3). If the alignment is incorrect, it should be specified whether the fixation is rotated (as shown in Figure 4) or inclined (as demonstrated in Figure 5) in various gaze positions.

Figure 5 – Inclined fixation   *Figure 4 – Rotated fixation*   *Figure 3 – Central fixation*

Functional Assessment Scale of Hemianopsia (FLASH) - User Guide

The final section of the scale addresses behaviors that may manifest during activities conducted in a neurorehabilitation setting. It is designed to assess the presence or absence of specific phenomena. For example, some items gauge whether patients employ head compensation strategies, such as tilting or rotating their heads to explore impaired visual fields. These strategies may lead to consistently omitting a well-defined portion of space, either to the right or left, during tasks like writing, despite having learned compensation techniques during rehabilitation. Patients with neglect, for instance, might be encouraged to identify a red line at the sheet's edge during tasks or to number lines to aid their spatial awareness.

The scale also includes items related to spatial management in motor activities, assessing deviations in trajectory, collisions with obstacles or people, and whether patients are surprised by sudden appearances of individuals or objects on their affected side.

The selection of items for the FLASH scale was based on the most common symptoms experienced by patients with hemianopia during their daily activities. Each item allows for indicating whether the phenomenon is present, absent, or not assessable (e.g., when a patient has not yet achieved the motor autonomy to move independently, the corresponding item will be marked as not assessable).

Finally, at the end of the scale, there is a designated space for recording any additional information not previously indicated.

Functional Assessment Scale of Hemianopia (FLASH)

Data

_____/_____/_____

Nome:

Cognome:

Diagnosi:

Professionista (Nome e cognome)

OSSERVAZIONE CLINICA QUALITATIVA

**a.** Direzione spontanea dello sguardo	☐ centrale	☐ Destra (dx)	☐ sinistra (sx)		
**b.** Posizione anomala della testa	☐ Si Ruotata a dx	☐ Si Ruotata a sx	☐ Si Ruotata a dx	☐ Si Ruotata a sx	☐ no
**c. Allineamento della fissazione binoculare**	☐ centrale	☐ Ruotata a dx	☐ Ruotata a sx	☐ Inclinata a dx	☐ Inclinata a sx

Firma

SETTING RIABILITATIVO

Utilizzo di strategie di compensazione della testa nelle prestazioni delle attività da scrivania	☐ si	☐ no	☐ N.A.
- e. In caso di risposta affermativa:			
Quando esplora un emicampo (destro/sinistro)	☐ si (specificare quale) ________________	☐ no	☐ N.A.
Quando esplora solo una parte dell'emicampo (ad esempio, il quadrante in basso a destra)	☐ si (specificare quale) ________________	☐ no	☐ N.A.
f. Nelle attività come la scrittura, la persona spesso inizia a utilizzare strategie di compensazione, anche quando sollecitato, e trascura costantemente una parte specifica dello spazio, che sia sul lato destro o sul lato sinistro.	☐ si	☐ no	☐ N.A.
g. Incontra ostacoli nel mantenere la posizione corretta della testa e/o dello sguardo.	☐ si	☐ no	☐ N.A.
h. Si osserva una deviazione dal percorso o dalla guida prevista della sedia a rotelle.	☐ si	☐ no	☐ N.A.
i. Si riscontrano difficoltà e disagi nel muoversi in aree affollate a causa dell'incontro con persone e vari ostacoli.	☐ si	☐ no	☐ N.A.
**l.** ruota la testa e dirige lo sguardo verso l'emicampo ridotto e talvolta sembra sorpreso quando identifica una persona o un oggetto che gli/le viene indicato.	☐ si	☐ no	☐ N.A.
m. sembra sorpreso/a dalla comparsa improvvisa di oggetti o persone nelle sue vicinanze.	☐ si	☐ no	☐ N.A.

NOTE

Functional Assessment Scale of Hemianopsia (FLASH) – guida all'uso

La scala è composta da due sezioni principali. La prima parte riguarda la registrazione di informazioni personali e cliniche del paziente, come il nome, il cognome e l'eziologia della loro malattia, insieme alla data di registrazione dell'osservazione. La seconda sezione è dedicata alle osservazioni cliniche qualitative. Comprende dati che emergono durante l'esame iniziale del paziente quando viene osservato per la prima volta. Questa sezione include osservazioni legate alla posizione della testa del paziente, alla direzione spontanea dello sguardo e alla presenza o assenza di deviazioni dello sguardo. Non vengono proposti test specifici; invece, ci basiamo sull'osservazione qualitativa. Ad esempio, nella sottosezione 'direzione spontanea dello sguardo, registriamo la direzione dello sguardo del paziente nella loro condizione di base, senza stimolazioni esterne.

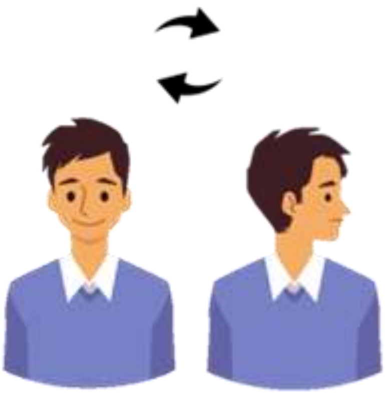

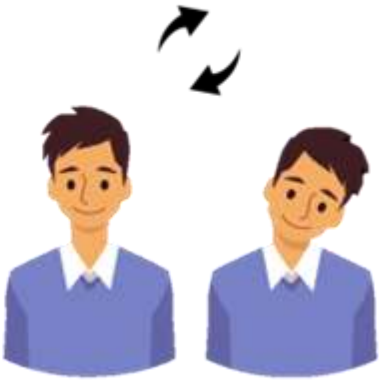

Una "Posizione anomala della testa" si riferisce a un orientamento obbligato della testa causato da anomalie che colpiscono il sistema visivo. Questa condizione è indicata dalla presenza di qualsiasi rotazione o inclinazione della testa. Di seguito, ci sono due immagini che possono aiutare a distinguere tra queste diverse condizioni posturali: rotazione (Figura 1) e inclinazione (Figura 2).

Figura 1- Rotazione

Figura 2- Inclinazione

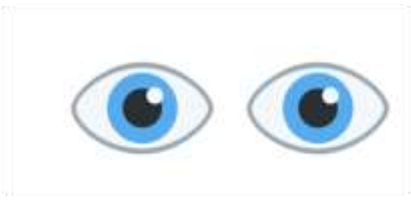

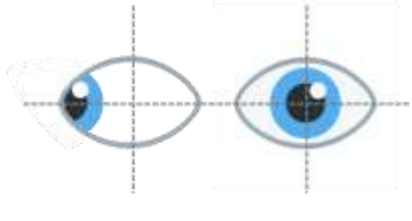

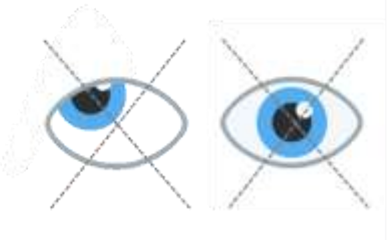

Nella sezione 'Allineamento della fissazione binoculare', si annoterà 'Fissazione centrale' quando gli assi visivi sono correttamente allineati (vedi Figura 3). Se l'allineamento è scorretto, dovrebbe essere specificato se la fissazione è ruotata (come mostrato in Figura 4) o inclinata (come dimostrato in Figura 5) in diverse posizioni dello sguardo.

Figura 3- Fissazione centrale

Figura 4- Fissazione ruotata

Figura 5- Fissazione inclinata

La sezione finale della Scala di Valutazione Funzionale dell'Emianopsia (FLASH) affronta i comportamenti che possono manifestarsi durante le attività svolte in un contesto di neuroriabilitazione. È progettata per valutare la presenza o l'assenza di fenomeni specifici. Ad esempio, alcuni elementi valutano se i pazienti utilizzano strategie di compensazione della testa, come inclinare o ruotare la testa per esplorare campi visivi compromessi. Queste strategie possono portare a tralasciare in modo costante una porzione ben definita dello spazio, sia a destra che a sinistra, durante attività come la scrittura, nonostante abbiano imparato tecniche di compensazione durante la riabilitazione. Ad esempio, i pazienti con la sindrome di negligenza potrebbero essere incoraggiati a individuare una linea rossa sul bordo del foglio durante le attività o a numerare le linee per aiutare la loro consapevolezza spaziale.

La scala include anche elementi legati alla gestione dello spazio nelle attività motorie, valutando deviazioni nella traiettoria, collisioni con ostacoli o persone e se i pazienti sono sorpresi dall'apparizione improvvisa di individui o oggetti sul lato affetto.

La selezione degli elementi per la scala FLASH si basa sui sintomi più comuni sperimentati dai pazienti con emianopia durante le loro attività quotidiane. Ciascun elemento consente di indicare se il fenomeno è presente, assente o non valutabile (ad esempio, quando un paziente non ha ancora raggiunto l'autonomia motoria per muoversi in modo indipendente, l'elemento corrispondente verrà contrassegnato come non valutabile).

Infine, alla fine della scala, c'è uno spazio designato per registrare eventuali informazioni aggiuntive non precedentemente indicate.
